# Spore-based arbuscular mycorrhizal fungal community in an olive cultivation area in southern Brazil

**DOI:** 10.1007/s00203-026-05139-3

**Published:** 2026-08-22

**Authors:** Arthur Joanello Cemin, Vagner Luiz Graeff-Filho, José Pedro Spies Nolibos, Ezequiel Cesar Carvalho Miola, Paulo Mello-Farias, Vanessa Sacramento Cerqueira

**Affiliations:** 1 Programa de Pós-Graduação em Sistema de Produção Agrícola Familiar, Departamento de Fitotecnia, Universidade Federal de Pelotas, Capão do Leão, RS Brazil; 2 Programa de Pós-Graduação em Fitossanidade, Universidade Federal de Pelotas, Capão do Leão, RS Brazil; 3 Programa de Pós-Graduação em Manejo e Conservação do Solo e da Água, Departamento de Solos, Universidade Federal de Pelotas, Capão do Leão, RS Brazil; 4 Programa de Pós-Graduação em Agronomia, Departamento de Fitotecnia, Universidade Federal de Pelotas, Capão do Leão, RS Brazil

**Keywords:** Arbuscular mycorrhizal fungi (AMF), Glomeromycota, Native AMF species, Olea europaea, Spore morphology

## Abstract

Arbuscular mycorrhizal fungi (AMF), belonging to the phylum Glomeromycota, establish symbiotic associations with approximately 90% of terrestrial plant species and play a key role in nutrient acquisition, tolerance to abiotic stresses, and agroecosystem functioning. Although olive cultivation has expanded in southern Brazil, information on native AMF communities associated with this crop remains scarce. This study characterized the AMF community associated with the rhizosphere of the olive cultivars Arbequina, Coratina, and Picual cultivated in Rio Grande do Sul, Brazil. Soil sampling was conducted in February 2025, during the summer dry season, when trees were in a vegetative growth, were not flowering or fruiting. Because AMF sporulation may vary according to host phenology and seasonal environmental conditions, the present survey reflects fungal communities under vegetative growth during the dry season. Spores were extracted by wet sieving and identified based on morphological characteristics. Six species belonging to five genera: *Acaulospora koskei, Glomus ambisporum, Diversispora globifera, Gigaspora decipiens, Gigaspora rosea,* and *Scutellospora calospora*. *G*. *ambisporum* was dominant in all cultivars. Differences among cultivars were observed for *G. decipiens, D. globifera, and G. rosea*. Cultivar identity explained 56.2% of the total compositional variation. Coratina showed lower evenness due to the dominance of *G. ambisporum*. SIMPER analysis indicated that *G. ambisporum* and *G. decipiens* accounted for 59.7% of the dissimilarity between Arbequina and Coratina. This study represents the first inventory of AMF associated with olive rhizospheres in southern Brazil and provides a basis for selecting native isolates for future inoculant development.

**Supplementary Information:**

The online version contains supplementary material available at https://doi.org/10.1007/s00203-026-05139-3.

## Introduction

Arbuscular mycorrhizal fungi (AMF), belonging to the phylum Glomeromycota, establish symbiotic associations with approximately 80–90% of terrestrial plant species (Wang et al. 2023; Wu et al. 2024). The evolutionary success of these fungi is largely attributed to their ability to form mutualistic interactions with plant roots in diverse ecosystems, contributing to their cosmopolitan distribution (Bonfante 2022; Rosling et al. 2024). Inside the root cortex cells, AMF develop highly branched structures called arbuscules, which constitute the main site of nutrient exchange between the fungus and the host plant (Prates Júnior et al. 2021). These fungi also produce vesicles that serve as lipid-rich storage structures, providing energy reserves for fungal development, while extracellular vesicles can contribute to molecular communication between plant and fungus (Holland and Roth 2023; Kameoka and Gutjahr 2022). Furthermore, extraradical hyphal networks extend beyond root depletion zones, expanding soil exploration and favoring the absorption of water and low-mobility nutrients such as phosphorus and zinc (Diagne et al. 2020; Hanane et al. 2020; Smith and Smith 2011).

In addition to their nutritional function, AMF contribute to plant tolerance to abiotic stresses, including drought, salinity, and heavy metal toxicity, promoting greater resilience in adverse environmental conditions (Dhalaria et al. 2020; Ennajeh and Ouledali 2024; Matos et al. 2022). Functional mycorrhizal associations have also been linked to better establishment and greater survival of transplanted seedlings, a critical factor for perennial agricultural systems (Ahmed et al. 2025; Meddad-Hamza et al. 2017). At the ecosystem level, AMF influence soil aggregation processes and structure rhizosphere microbial communities, contributing to the maintenance of biodiversity and the functioning of ecosystems (Shi et al. 2022; Shukla et al. 2025).

However, the composition and sporulation dynamics of AMF communities are also influenced by the plant species and the phenological stage of the host (Coelho et al. 2024; Deepika and Kothamasi 2021; Wang et al. 2024). Previous studies have shown that spore abundance and root colonization may vary throughout plant development, with higher sporulation frequently observed during reproductive and maturation stages, whereas the flowering stage may be associated with reduced spore production due to the high phosphorus demand of the host plant (Bohacz et al. 2022; Coelho et al. 2024). Furthermore, environmental management and edaphoclimatic characteristics such as seasonal fluctuations in precipitation and temperature also strongly affect AMF dynamics (Dong et al. 2025; Sakha et al. 2025). In tropical and subtropical environments, dry periods are generally associated with higher spore densities, under conditions of water limitation and elevated soil temperatures, while during the rainy seasons the concentration of spores is low and soil moisture limits colonization and symbiosis but not the viability of AMF (Coelho et al. 2024; Tenzin et al. 2022). Thus, the characterization of native AMF communities has been increasingly used as an ecological indicator of soil health and agroecosystem functioning (Bohacz et al. 2022).

In the context of olive growing, the plant benefits from mycorrhizal association through increased nutrient uptake and greater tolerance to environmental stresses (Chenchouni et al. 2020; Robã et al. 2024; Tekaya et al. 2022). Olive cultivation in Brazil has been increasing due to its economic potential and benefits for human health, which contain bioactive compounds that help prevent cardiovascular disease and diabetes (Bucciantini et al. 2021; Dos Santos et al. 2023; Sá 2024). Brazil has approximately 4,266 ha devoted to olive cultivation, with an average productivity of 1,054.6 kg ha^−1^ of olives (FAOSTAT 2024). Rio Grande do Sul is currently the leading olive-producing state in the country, generating more than BRL 10.462 million in production value (IBGE 2024). The expansion and consolidation of olive cultivation in the state have been fostered by initiatives such as the Programa Estadual de Desenvolvimento da Olivicultura (PRÓ-OLIVA), among the most widely cultivated olive cultivars in Rio Grande do Sul are Arbequina, Coratina, and Picual, which differ in key agronomic traits: Arbequina is a Spanish cultivar with early bearing and wide climatic adaptability; Coratina is an Italian cultivar valued for its high polyphenol content and antioxidant activity; and Picual is the world's most widely cultivated Spanish cultivar, known for its high oil yield and cold tolerance (Coutinho et al. 2009; Grompone and Villamil 2013; Miho et al. 2021). which promotes the adoption of technologies aimed at olive and olive oil production, contributing to increased productivity and enhanced income for family farmers through the establishment of new orchards (SEAPI 2025). However, the success of implementing and producing new orchards depends on the use of fertilizers, which contributes to Brazil's economic dependence on importing 85% of the mineral fertilizers used in the country (Al-Din and Alalaf 2025; Roma et al. 2023; Russo and Figueira 2023).

Given this scenario, the use of bioinputs such as arbuscular mycorrhizal fungi emerges as a strategic alternative to reduce dependence on chemical fertilizers (Ouhaddou et al. 2025), contributing to a more sustainable and accessible production model for family farming.

However, for these bioinputs to be effective, it is necessary to develop inoculants adapted to local conditions, since the composition of AMF communities is strongly influenced by edaphoclimatic factors, agricultural management, and plant species (Davison et al. 2021; Frew et al. 2025; Robã et al. 2024). Thus, characterizing the species naturally associated with established crops represents an essential first step in the development of bioinputs. Furthermore, the recovery of AMF spores from the rhizosphere may indicate the presence of infectious propagules and reflect the sporulating fraction of the community, suggesting that the taxa present constitute potential symbiotic partners for the host plants, although spore occurrence does not directly indicate active root colonization (da Silva et al. 2022; Kebede et al. 2023; Sivakumar 2013). No published data on root colonization rates in olive plantations within the study area are currently available, which further highlights the relevance of the present study as a baseline characterization.

In this context, it is hypothesized that the genotype of olive cultivars acts as a determining ecological filter in the assemblage of arbuscular mycorrhizal fungal communities, modulating not only the taxonomic composition but also the dominance structure and functional spectrum of associated taxa, with potential implications for symbiosis efficiency and nutrient acquisition. The objectives of this study were to characterize the arbuscular mycorrhizal fungi community associated with the rhizosphere of three olive cultivars cultivated in southern Brazil, to determine spore density and diversity indices for each cultivar, and to evaluate whether cultivar identity influences AMF community composition. In addition, this study provides the first inventory of AMF associated with olive orchards in Rio Grande do Sul, based on samples collected at the Palma Agricultural Center of the Federal University of Pelotas (UFPel). This study represents a first step towards future investigations aimed at elucidating the diversity and ecological functioning of arbuscular mycorrhizal fungi (AMF) communities in olive agroecosystems. Furthermore, knowledge of the native AMF community could support future studies focused on the development of regionally adapted bioinoculants and other mycorrhiza-based bioinputs.

## Materials and methods

### Study area, soil sampling and collection

**Fig. 1:**
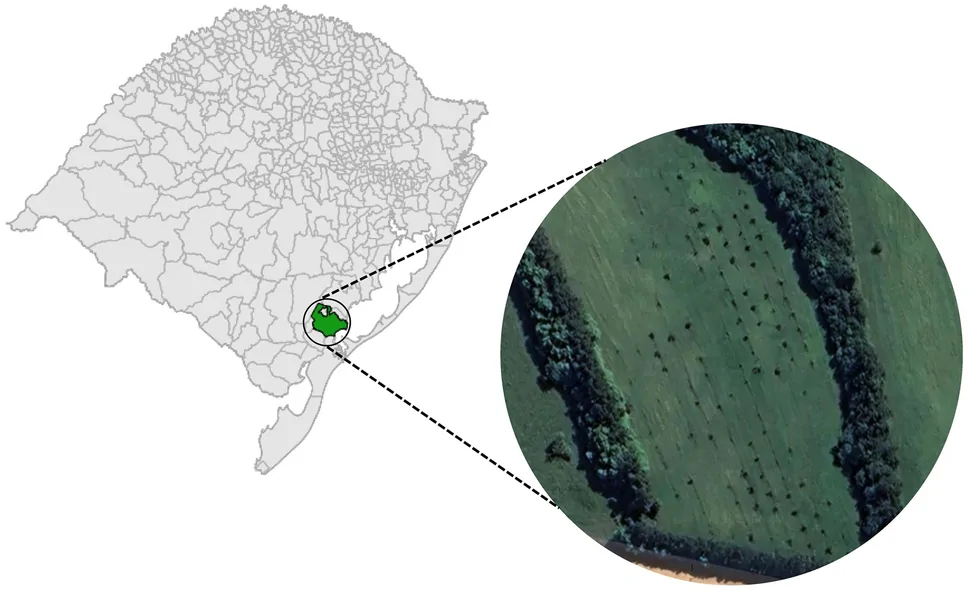
Geographic location of the study area. The map shows the location of Rio Grande do Sul in southern Brazil and a zoomed view of the experimental olive orchard at Centro Agropecuário da Palma, Universidade Federal de Pelotas, where the olive cultivars sampled in this study are located

The study was conducted at the Palma Agricultural Center of the Federal University of Pelotas (UFPel) (31.8031° S, 52.5082° W), located in Capão do Leão, Rio Grande do Sul, Brazil (Fig. 1). The climate is humid subtropical, characterized by hot summers and cold winters (Kuinchtner and Buriol 2001). The study area is situated in a transitional zone between the Pampa Biome and the Atlantic Forest, being characterized by a mosaic landscape composed of herbaceous-shrubby grasslands of the Coastal Plain and remnants of Seasonal Semideciduous Forest distributed over the slopes of the Sul-Riograndense Shield. Wetlands and marshes are also common elements of the regional landscape (IBGE 2019; Venzke 2012).

The experimental olive orchard was nine years old and consisted of several olive cultivars grown under the same environmental conditions, with each planting row corresponding to a single cultivar. Three cultivars, namely Arbequina, Coratina, and Picual, were selected for this study because they are among the most representative olive cultivars cultivated in Rio Grande do Sul, occurring in 97%, 30%, and 75% of orchards, respectively (Oliveira et al. 2022), and because they exhibit contrasting agronomic and biochemical characteristics (Coutinho et al. 2009; Grompone and Villamil 2013; Miho et al. 2021). The orchard has been managed under a rainfed, low-input system for the past two years, with no application of synthetic fertilizers, herbicides, or pesticides during this period. At the time of sampling, the trees were in the vegetative growth stage, and spontaneous vegetation, predominantly composed of grasses (Poaceae), was present between rows.

For each cultivar, four trees were randomly selected, totaling twelve sampled trees. To minimize edge effects, only the central trees of each row were considered for sampling. Rhizospheric soil was collected from each tree at a depth of 0–20 cm. Three subsamples were taken at equidistant points around the root zone of each tree after removing the litter layer and were subsequently homogenized to form one composite sample per tree, resulting in four independent composite samples for each cultivar (n = 4), totaling 12 experimental samples. From each composite sample, 100 g of soil were used for spore extraction. Since the three cultivars were grown within the same cultivation system, the remaining soil from the twelve composite samples was pooled and homogenized to obtain a representative sample of the experimental orchard, which was used for the determination of soil macro- and micronutrient contents (Table 1).

**Table 1:** Physicochemical characterization of the soil in the olive cultivation area of the Palma Agricultural Center

Category	Nutrient/Parameter	Value	Unit
pH	pH in water (1:1)	5.4	
Macronutrients	Calcium (Ca)	3.1	cmolc/dm^3^
	Magnesium (Mg)	1.1	cmolc/dm^3^
	Potassium (K)	0.15	cmolc/dm^3^
	Phosphorus (P–Mehlich)	9.3	mg/dm^3^
	Sulfur (S–SO_4_)	6.9	mg/dm^3^
	Organic matter (OM)	1.66	%
Micronutrients	Zinc (Zn)	1.2	mg/dm^3^
	Copper (Cu)	1.2	mg/dm^3^
	Manganese (Mn)	59	mg/dm^3^
	Sodium (Na)	9	mg/dm^3^
	Iron (Fe)	0.16	%

### Spore extraction and identification

Arbuscular mycorrhizal fungi spores were extracted from each sample using the wet sieving method (Gerdemann and Nicolson 1963), by agitating 100 g of soil in 2 L of water, followed by decantation and passage through 0.84 mm, 0.42 mm, and 0.149 mm sieves, repeating the process three times. The material retained on the 0.149 mm sieve was transferred to 50 mL Falcon tubes and centrifuged at 2,000 rpm for 3 min. The supernatant was discarded, and the sediment was resuspended in a 50% (w/v) sucrose solution, followed by a second centrifugation at 2,000 rpm for 2 min. The supernatant, containing the floating spores, was immediately passed through a 0.149 mm sieve and washed with distilled water. Spores were transferred to Petri dishes and counted under a stereomicroscope. Then, the spores were carefully separated into morphotypes based on size and color and mounted on slides using PVLG (polyvinyl-lactoglycerol) and PVLG mixed with Melzer's reagent (1:1), as described by Koske and Tessier (1983). The slides were kept at room temperature for about a week and subsequently dried in an oven at 35 °C for 3 days. The slides were inspected under a light microscope and identified to genus and/or species level based on the reaction of the spore wall structure to Melzer's reagent, and compared with descriptions from the pages of the International Collection of Arbuscular Mycorrhizal Fungi (INVAM) at the University of Kansas, USA (INVAM 2024).

## Statistical analysis

### Frequency of occurrence and spore abundance

The frequency of occurrence (FO%) of each arbuscular mycorrhizal fungal species was calculated as the percentage of sampling units in which the species was present relative to the total number of sampling units (n = 12). Species frequency was determined according to the following equation:

$$ \begin{gathered} {\mathrm{FO}}\left( \% \right) \hfill \\ = \left( {{\mathrm{number}}\;{\mathrm{of}}\;{\mathrm{sampling}}\;{\mathrm{units}}\;{\mathrm{in}}\;{\mathrm{which}}\;{\mathrm{the}}\;{\mathrm{species}}\;{\mathrm{occurred}}/{\mathrm{total}}\;{\mathrm{number}}\;{\mathrm{of}}\;{\mathrm{sampling}}\;{\mathrm{units}}} \right) \hfill \\ \times {1}00. \hfill \\ \end{gathered} $$

Differences in spore abundance among AMF species and olive cultivars were evaluated using generalized linear mixed models (GLMMs) with a negative binomial distribution (Brooks et al. 2017). Cultivar, AMF species, and their interaction were included as fixed effects, whereas sampling units were treated as a random effect. Model adequacy was assessed using residual diagnostics (Hartig 2026), and multiple comparisons were performed using estimated marginal means with appropriate adjustment for multiple testing (Lenth 2026).

## Alpha diversity indices

Alpha diversity was evaluated for each sampling unit (n = 12) using the vegan package (Oksanen et al. 2026). Two diversity indices were calculated: the Shannon–Wiener index (H') and the Simpson index (D). Differences among olive cultivars were evaluated using generalized linear mixed models (GLMMs) fitted with the glmmTMB package (Brooks et al. 2017), with cultivar identity included as a fixed effect. The Shannon index was modeled assuming a Gaussian distribution, whereas the Simpson index was modeled assuming a beta distribution with logit link after transformation of the original values to avoid boundary values of 0 and 1. Pairwise comparisons among cultivars were performed using Tukey-adjusted estimated marginal means obtained with the emmeans package (Lenth 2026).

### Community composition and beta diversity

Beta diversity was assessed using the Bray–Curtis dissimilarity index calculated from spore abundance data. Differences in AMF community composition among olive cultivars were tested by permutational multivariate analysis of variance PERMANOVA with 999 permutations (Anderson 2001). Homogeneity of multivariate dispersion among cultivars was verified prior to PERMANOVA. Community structure was visualized by non-metric multidimensional scaling (Kruskal 1964). All multivariate analyses were performed using the vegan package (Oksanen et al. 2026).

The contribution of individual AMF species to the dissimilarity among olive cultivars was evaluated using similarity percentage analysis (SIMPER; Clarke 1993). Results are expressed as the percentage contribution of each species to the Bray–Curtis dissimilarity between cultivars. Analyses were performed using the vegan package (Oksanen et al. 2026).

## Results

### AMF species composition and frequency of occurrence

**Fig. 2:**
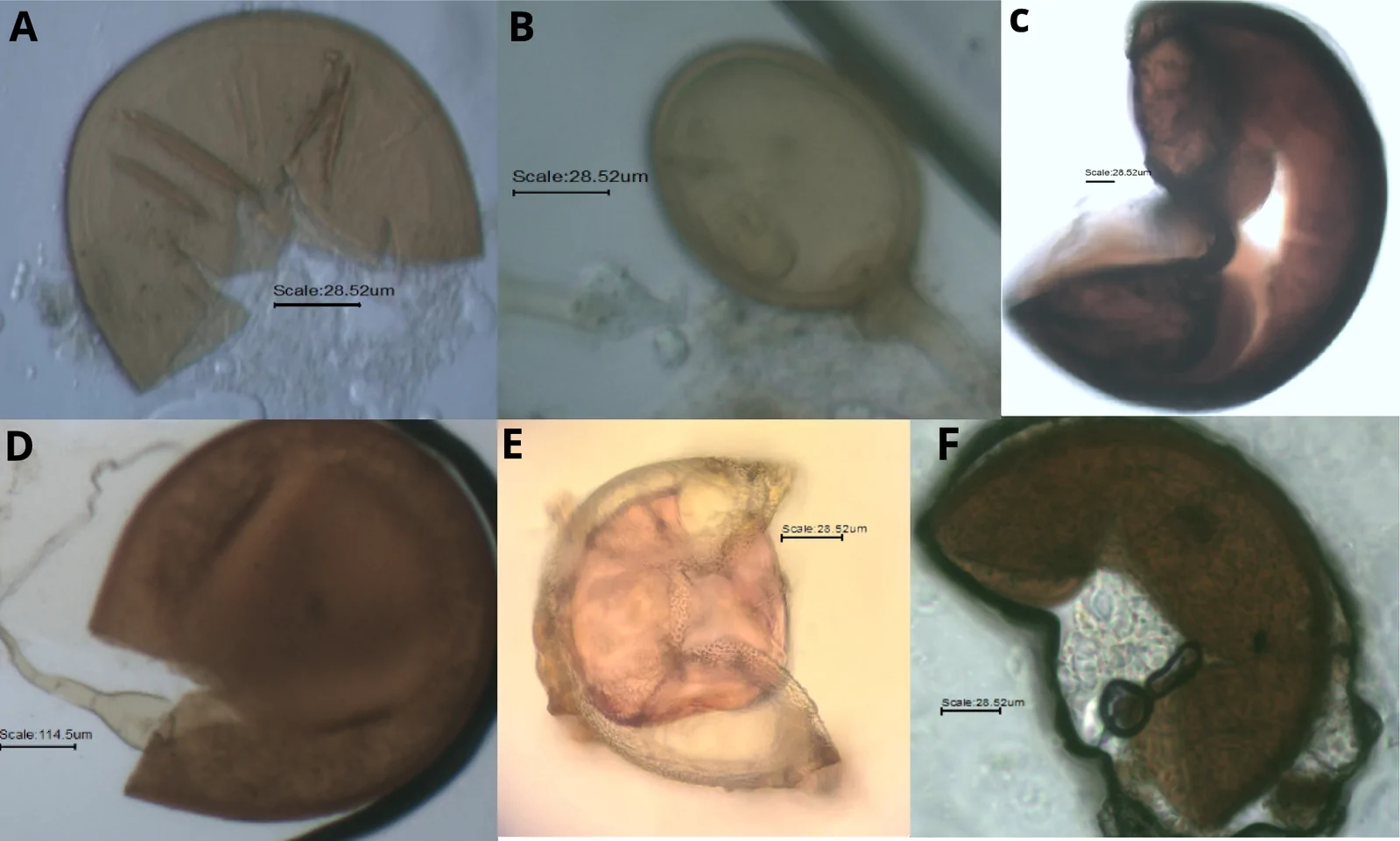
Optical microscopy images of spores of arbuscular mycorrhizal fungi (AMF) extracted from the rhizosphere soil of olive trees (*Olea europaea* L.) cultivated at the Centro Agropecuário da Palma, Universidade Federal de Pelotas, Rio Grande do Sul, Brazil. All spores were mounted on PVLG and PVLG + Melzer's reagent (1:1) and photographed using optical microscopy. **A***Acaulospora koskei.* Scale bar: 28.52 µm. **B***Diversispora globifera.*Scale bar: 28.52 µm. **C***Gigaspora decipiens.* Scale bar: 28.52 µm. **D***Gigaspora rosea.* Scale bar: 114.5 µm. **E** S*cutellospora calospora.* Scale bar: 28.52 µm. **F***Glomus ambisporum.* Scale bar: 28.52 µm

In the rhizosphere of the three olive cultivars, spores of six species belonging to five genera of four families of arbuscular mycorrhizal fungi (AMF) were found. The species found were *Acaulospora koskei* Błaszk, *Diversispora globifera* (Koske & C. Walker) C. Walker & A. Schüßler, *Gigaspora decipiens* I.R. Hall & L.K. Abbott, *Gigaspora rosea* (T.H. Nicolson & N.C. Schenck), *Scutellospora calospora* (T.H. Nicolson & Gerd.) C. (Walker & F.E. Sanders), and *Glomus ambisporum* G.S. Sm. & N.C. Schenck. (Fig. 2). The frequency of occurrence *(*FO*)* revealed that four species*, Glomus ambisporum, G. decipiens, G. rosea,* and *Di. globifera,* were present in all sampling units (FO = 100%), characterizing them as stable and generalist components of the mycorrhizal community associated with the studied olive cultivars. In contrast, *S. calospora* showed a more restricted distribution (FO = 25.0%), occurring only in samples from the Arbequina and Picual cultivars. Acaulospora koskei exhibited the lowest frequency (FO = 16.7%), being detected exclusively under the Picual cultivar (Table 2).

**Table 2:** Mean abundance (spores/100 g of soil ± SD) and frequency of occurrence (FO%) of AMF species by cultivar

Species	Arbequina	Coratina	Picual	FO (%)
*Glomus ambisporum*	58.2 ± 6.3	87.8 ± 22.2	77.5 ± 16.9	100
*Gigaspora decipiens*	39.0 ± 9.4	11.8 ± 2.4	20.0 ± 9.0	100
*Gigaspora rosea*	21.5 ± 2.9	17.2 ± 9.2	41.8 ± 20.5	100
*Diversispora globifera*	16.5 ± 8.2	38.8 ± 6.9	30.8 ± 10.1	100
*Scutellospora calospora*	8.0 ± 9.6	0.0 ± 0.0	2.2 ± 4.5	25.0
*Acaulospora koskei*	0.0 ± 0.0	0.0 ± 0.0	8.4 ± 12.3	16.7

### Spore abundance among cultivars

**Fig. 3:**
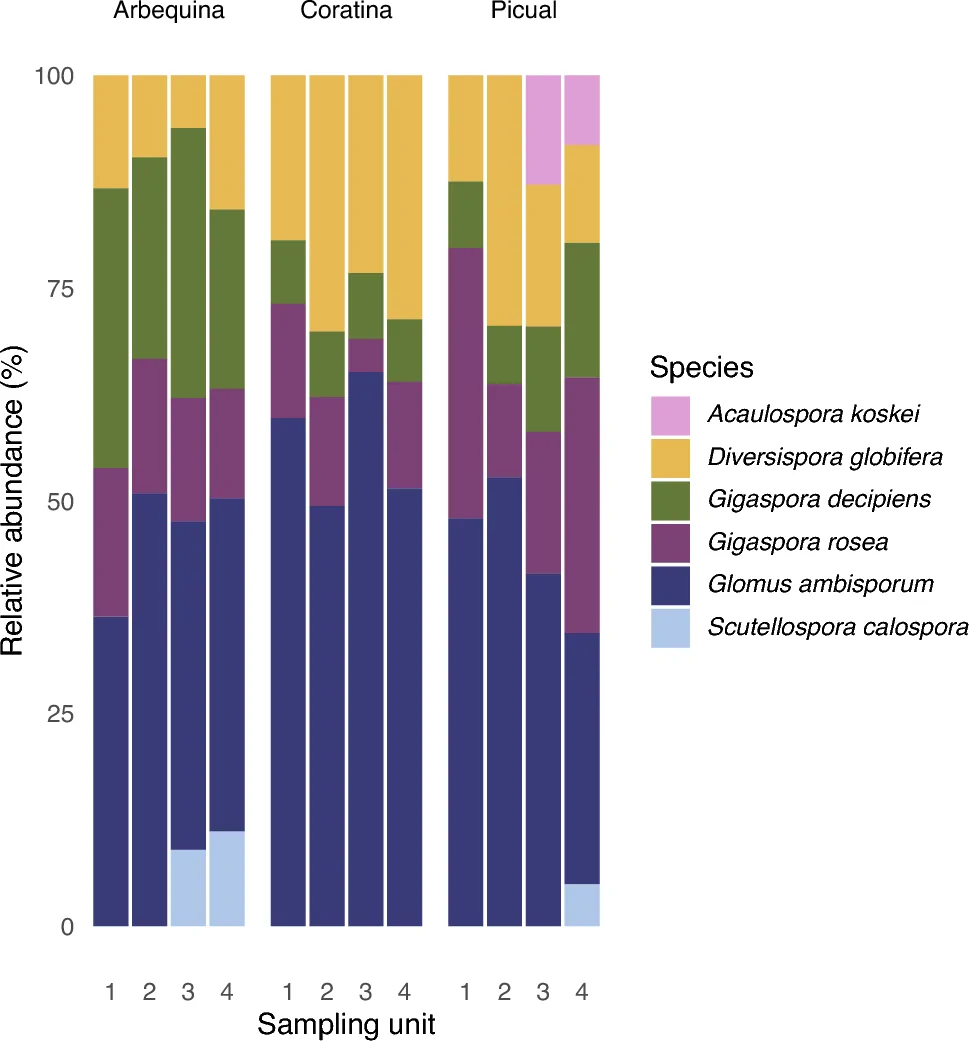
Stacked bar charts showing the relative abundance (%) of arbuscular mycorrhizal fungi (AMF) species per individual sampling unit, grouped by olive cultivar. The horizontal axis represents individual sampling units numbered 1–4 within each cultivar group (Arbequina, Coratina, and Picual, from left to right). The vertical axis represents relative abundance from 0 to 100%. Each color within the stacked bars corresponds to one of the six identified AMF species: *Acaulospora koskei* (lightest shade, pink), *Diversispora globifera* (medium purple), *Gigaspora decipiens* (dark green), *Gigaspora rosea* (medium green), *Glomus ambisporum* (dark purple), and *Scutellospora calospora* (light lilac). In all cultivars, *Glomus ambisporum* occupies the largest proportion of the bar

In all cultivars, *G. ambisporum* was the most abundant species (Fig. 3). In Arbequina, *G. ambisporum* exhibited 198% higher sporulation than *S. calospora* (*p* < 0.0001) and 126% higher sporulation than *D. globifera* (*p* = 0.005), with a marginal difference of approximately 100% compared with *G. rosea* (*p* = 0.050). In addition, *G. decipiens* showed 158% higher abundance than *S. calospora* (*p* = 0.0004), whereas no significant differences were detected among the remaining species. In Coratina, *G. ambisporum* was 201% more abundant than *G. decipiens* (*p* < 0.0001) and 163% more abundant than *G. rosea* (*p* < 0.0001). Furthermore, *D. globifera* exhibited 119% higher abundance than *G. decipiens* (*p* = 0.014). In Picual, *G. ambisporum* showed 354% higher sporulation than *S. calospora* (*p* < 0.0001), 136% higher sporulation than *G. decipiens* (*p* = 0.002), and 222% higher sporulation than *A. koskei* (*p* < 0.0001). Comparisons involving *A. koskei *and *S. calospora* among cultivars should be interpreted with caution because of their extremely low abundance and the associated high standard errors, resulting in non-significant comparisons (*p* = 1.0000).

The cultivar identity changed significantly in the abundance of three species (Fig. 3). *G. decipiens* showed a significantly higher spore abundance in Arbequina than in Coratina, with Arbequina showing approximately 232% higher abundance (*p* = 0.003). As for *D. globifera*, its abundance was significantly higher in Coratina compared to Arbequina, with Arbequina showing about 57% lower abundance (*p* = 0.045). In addition, the species *G. rosea* showed significantly lower abundance in Coratina than in Picual of approximately 59% (*p* = 0.035). No significant differences were blocked between cultivars for *G. ambisporum* (all *p* > 0.44).

### Diversity indices and multivariate analysis

**Fig. 4:**
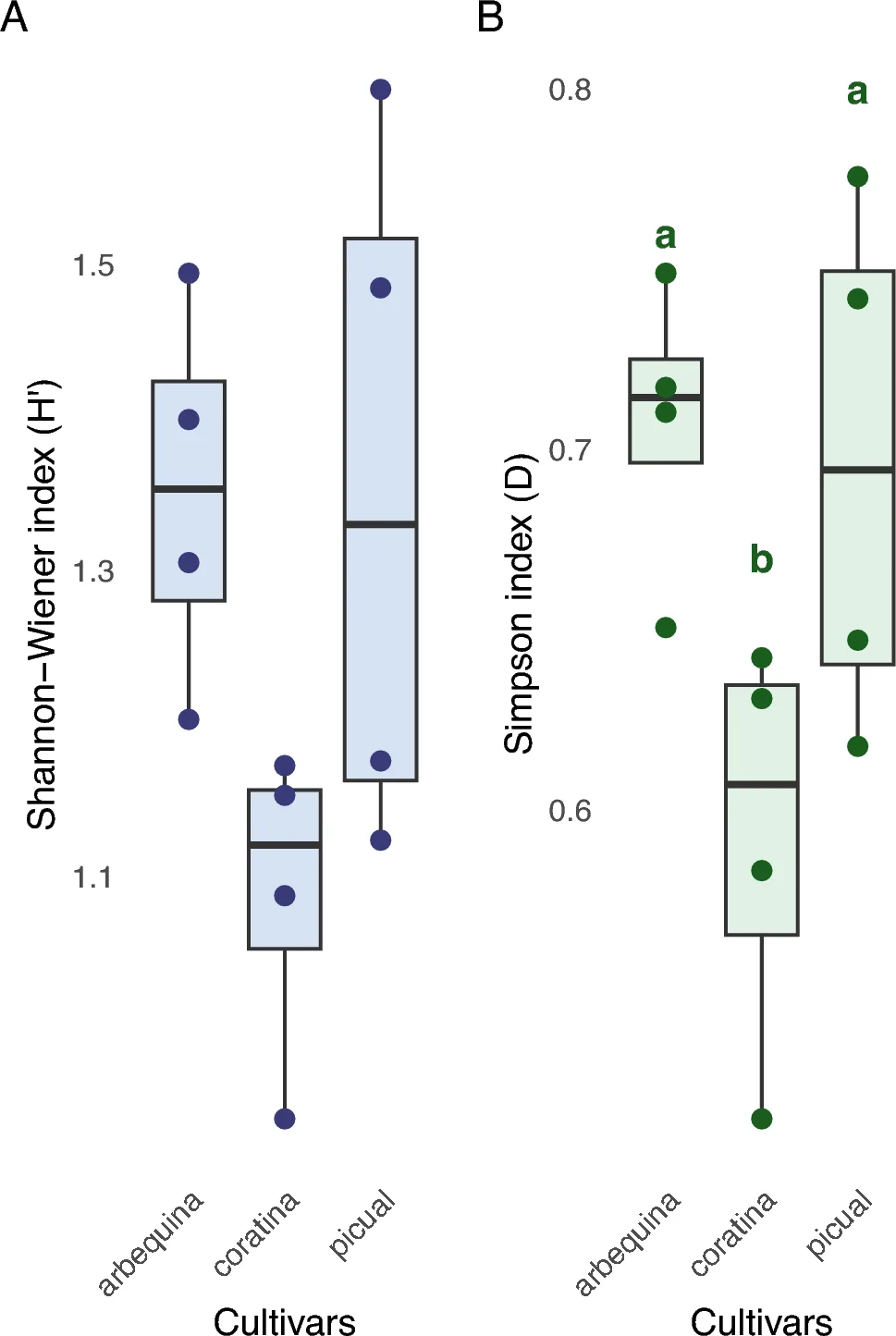
Graph containing two box plot graphs **A** Shannon–Wiener diversity index (H') per sampling unit grouped by cultivar (Arbequina, Coratina, Picual). Each point represents one sampling unit. Coratina shows lower and less variable H' values compared to Arbequina and Picual, though differences were not statistically significant, **B** Simpson dominance index (D) per sampling unit grouped by cultivar. The vertical axis ranges from approximately 0.5 to 0.8. Coratina showed significantly lower evenness compared to Arbequina and Picual indicated by the letter "b" below Coratina points and letter "a" above Arbequina and Picual points

The Shannon index (Fig. 4a) showed a non-significant marginal trend, with Coratina exhibiting lower values than Arbequina (Tukey *p* = 0.076; H' means: Arbequina 1.351 ± 0.125; Coratina 1.089 ± 0.103; Picual 1.350 ± 0.238). The Simpson index (Fig. 4b) showed that the Coratina cultivar presented significantly lower evenness than Arbequina (Tukey *p* = 0.006; means: 0.593 ± 0.059 vs. 0.707 ± 0.041) and Picual (*p* = 0.014; D = 0.696 ± 0.074), while Arbequina and Picual did not differ from each other (*p* = 0.968).

**Fig. 5:**
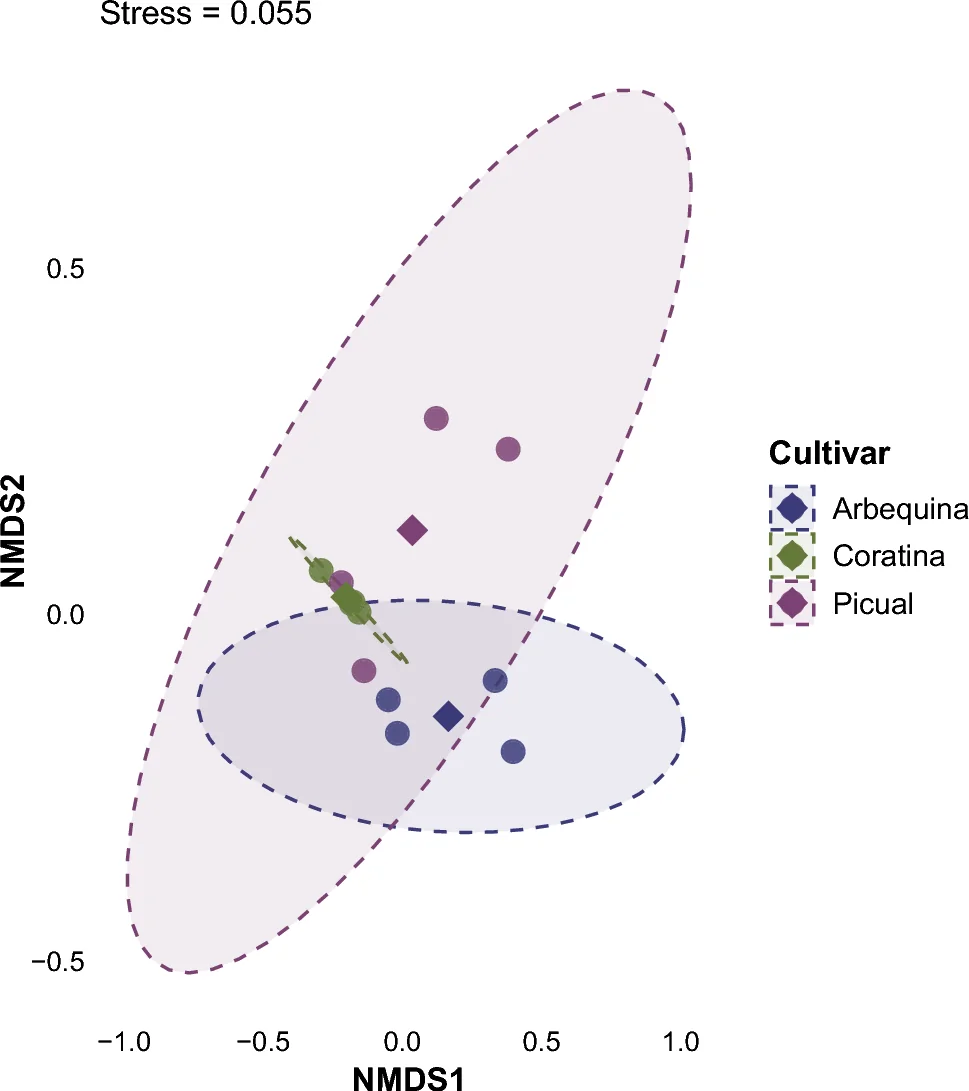
Ordination by non-metric multidimensional scaling (NMDS) based on Bray–Curtis dissimilarity among rhizosphere sampling units of olive trees (n = 12). Points represent individual sampling units; diamonds represent cultivar centroids; ellipses delimit the 95% confidence interval per group. Stress = 0.055

Multivariate analysis (Fig. 5) revealed that cultivar identity explained 56.2% of the total variation in AMF community composition (PERMANOVA: F2,9 = 5.772; R^2^ = 0.562; *p* = 0.004). The homogeneity of the multivariate dispersion was confirmed (betadispersion: F2,9 = 1.774; *p* = 0.224). NMDS ordination (stress = 0.055) visually corroborated the separation between cultivars, with Coratina forming the most cohesive internal cluster (Fig. 5). Paired PERMANOVA indicated that Arbequina differed significantly from Coratina (F = 12.875; R^2^ = 0.682; *p* = 0.033) and from Picual (F = 4.956; R^2^ = 0.452; *p* = 0.033), while Coratina and Picual did not differ significantly from each other (F = 2.666; R^2^ = 0.308; *p* = 0.154).

### Species contribution to dissimilarity

**Fig. 6:**
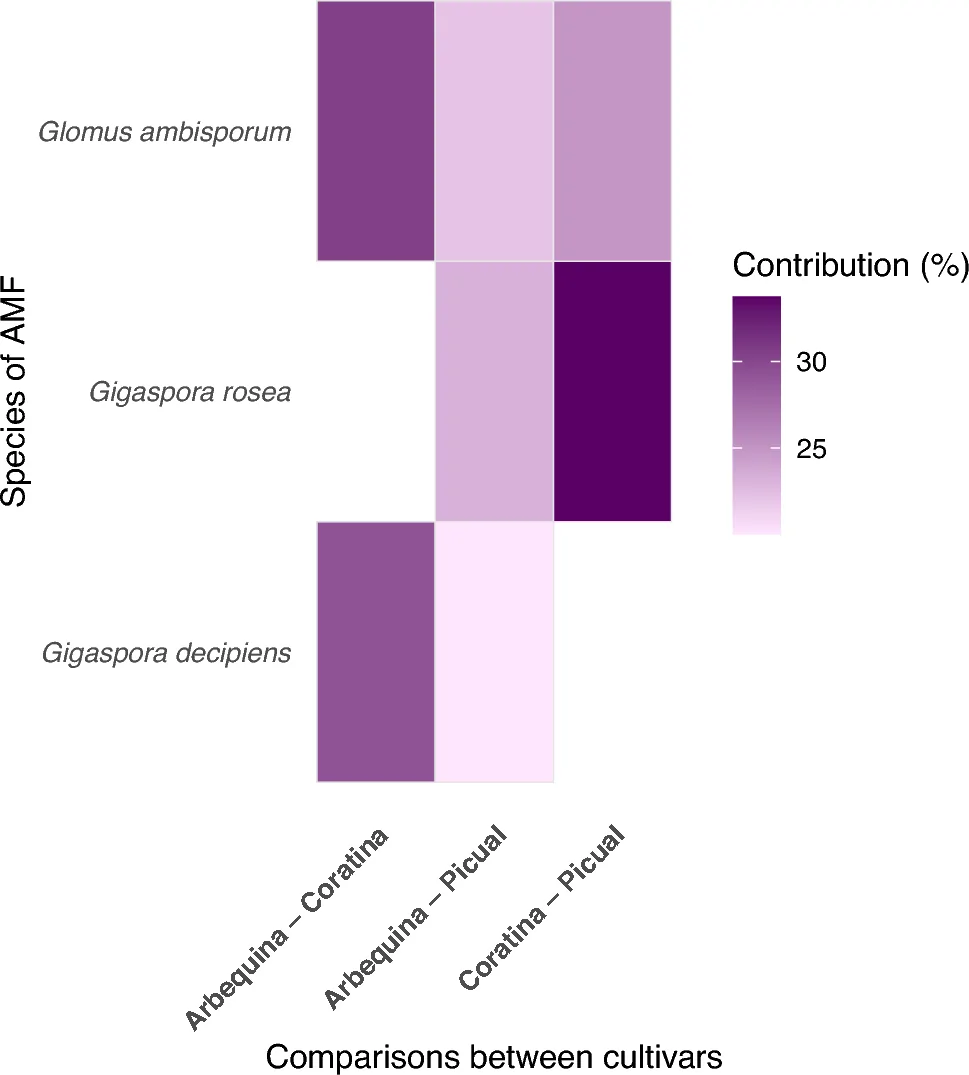
Contribution of arbuscular mycorrhizal fungi (AMF) species to Bray–Curtis dissimilarity between olive cultivar pairs, based on SIMPER analysis. Color intensity indicates the percentage contribution of each species to the dissimilarity of the respective cultivar pair. *Glomus ambisporum* and *Gigaspora decipiens* were the primary drivers of dissimilarity between Arbequina and Coratina, while *Gigaspora rosea* was the main contributor to the Coratina and Picual contrast

The SIMPER analysis (Fig. 6) is organized by the main species responsible for the dissimilarity between cultivars. In contrast to Arbequina and Coratina, *G. ambisporum* (30.4%; *p* = 0.016) and *G. decipiens* (cumulative contribution of 59.7%;* p* = 0.002) were the main contributors, followed by D. globifera (cumulative 83.9%; *p* = 0.007). In the Coratina–Picual contrast, *G. rosea* accounted for 33.7% of the dissimilarity (*p* = 0.026). No species reached statistical significance in the Arbequina–Picual contrast, a result consistent with the non-significant paired PERMANOVA for this pair.

## Discussion

This was the first study on the arbuscular mycorrhizal fungi community in the rhizosphere of olive trees in Rio Grande do Sul. The data obtained from the collection of rhizosphere soil from the three olive cultivars studied Arbequina, Coratina, and Picual allowed for the characterization of the autochthonous AMF species associated with olive cultivation at the region Centro Agropecuário da Palma (UFPel), establishing an initial ecological basis for understanding the composition and structure of AMF communities associated with olive cultivation in southern Brazil.

Species-level identification revealed a community composed of the genera *Glomus*, *Diversispora*, *Acaulospora*, *Gigaspora*, and *Scutellospora*, groups frequently reported in perennial agricultural systems and previously associated with olive cultivation, as reported by Calvente et al. (2004), Kachkouch et al. (2012), and Montes-Borrego et al. (2014). The species richness observed was higher than that reported by Calvente et al. (2004), who evaluated olive orchards under Mediterranean conditions in southern Spain, and similar to that found by Melloni et al. (2020), who investigated multiple olive cultivars grown in clay soils under subtropical conditions in Brazil. In contrast, slightly higher diversity values were observed in the study by Ouledali et al. (2022), conducted in southern Tunisia along an aridity gradient, and Meddad-Hamza et al. (2017) conducted in Algeria with a large climatic distance from humidity to desert. These differences among studies may suggest that regional edaphoclimatic conditions and soil properties influence the structure of AMF communities in olive orchards. Variations in temperature and precipitation can affect sporulation patterns and species composition (Davison et al. 2021), whereas phosphorus availability and soil organic matter content are important determinants of AMF diversity. High phosphorus levels tend to reduce both root colonization and sporulation, whereas higher organic matter contents may promote microbial activity and enhance fungal diversity (Berruti et al. 2016; Kemmelmeier et al. 2022; Rui et al. 2022). Therefore, differences in climatic conditions and soil chemical characteristics likely contributed to the variation observed among studies.

Comparisons with AMF communities associated with native vegetation provide additional ecological context for interpreting the patterns observed in the olive orchards. Studies conducted in native grassland areas with sandy soils of the Pampa biome in southern Brazil reported the occurrence of the genera *Glomus, Acaulospora, Gigaspor*a, and *Scutellospora*, including the species *Acaulospora scrobiculata, Gigaspora margarita, Glomus clarum* and *Scutellospora heterogama* (Mello et al. 2006). Similar patterns were described in temperate grasslands of the Flooded Pampa biome in Argentina, where species of the genera Glomus and Acaulospora were reported (Escudero and Mendoza 2005). Although direct comparisons are limited by differences in host species and environmental conditions, the recurrent occurrence of some of these genera in both native ecosystems and olive orchards suggests that part of AMF community associated with olive trees may be derived from the regional species pool of the Pampa biome. However, some species recorded in the present study, such as *G. ambisporum, D. globifera, G. decipiens,* and *G. rosea*, were not reported in surveys conducted in natural Pampa areas, which may indicate that olive cultivation and the specific conditions of the agricultural environment influenced the composition of these fungi. Therefore, future studies incorporating direct comparisons between olive orchards and adjacent native vegetation are essential to test this hypothesis and understand whether agricultural shifts reshape autochthonous mycorrhizal communities.

The predominance of *G. ambisporum* in all cultivars, surpassing other species such as *G. decipiens*, *D. globifera*, and *G. rosea*, is consistent with literature. Similar patterns of dominance of the genus Glomus in olive groves in morphological and molecular characterization studies have already been widely documented (Bizos et al. 2020; Montes-Borrego et al. 2014; Palla et al. 2020). In Brazil, Melloni et al. (2020) also reported *G. ambisporum* as the most abundant arbuscular mycorrhizal fungal species associated with olive trees. This dominance pattern may be related to the high ecological adaptability of species in the Glomeraceae family, which exhibit greater tolerance to different agricultural management practices (Ouhaddou et al. 2025; Zhang et al. 2021) and adverse environmental conditions, including saline soils and more arid environments (Ouledali et al. 2022; Malik et al. 2025).

A possible explanation for the greater persistence of these species in agroecosystems is associated with their functional characteristics. Members of the Glomeraceae family exhibit ruderal characteristics, such as high sporulation capacity, rapid renewal of extraradical mycelium, the ability to allocate biomass within the root, and are able to colonize new hosts through hyphal fragments present in the soil (Cahyaningtyas and Ezawa 2024; Zhang et al. 2021). This set of strategies favors the rapid recolonization of the root system and contributes to the maintenance of these species in agricultural matrices. The prominence of *Glomus* in this inventory may suggest an evolutionary adaptation to managed perennial systems, leading to representatives of Glomeraceae being frequently reported as dominant in studies characterizing arbuscular mycorrhizal fungi in agricultural environments. However, further controlled and longitudinal studies are required to experimentally test this hypothesis and confirm the underlying mechanisms driving this adaptation in olive orchards. 

In contrast, the occurrence of species belonging to the Gigasporaceae family, such as *G. decipiens, G. rosea,* and *S. calospora*, contributed to the functional diversity of the community, despite their low relative abundances. Their lower abundance may be related to the ecological strategy typically exhibited by members of this family, which allocate a substantial proportion of their biomass to the development of extensive extraradical hyphal networks and producing spores only in later stages of the life cycle (Arcidiacono et al. 2024; Cahyaningtyas and Ezawa 2024). In addition, these fungi preferentially colonize new hosts through intact hyphal networks rather than fragmented hyphae, which may reduce their persistence under managed agricultural conditions (Cahyaningtyas and Ezawa 2024; Dhumal and Shinde 2020; Johnny et al. 2025). Despite their lower abundance, species of this family are functionally important because they contribute to soil exploration and the acquisition of poorly mobile nutrients (Arcidiacono et al. 2024), which could increase the functional diversity of AMF communities in olive orchards.

Although climatic and physicochemical variables of the soil were not directly evaluated, previous studies demonstrate that pH, temperature, and humidity are determining factors in the structuring of AMF communities in olive groves, functioning as ecological filters for the species (Montes-Borrego et al. 2014; Meddad-Hamza et al. 2017). The soil pH observed in this study (5.4) likely acted as an important environmental filter, influencing the composition of the detected species, since variations in this parameter are frequently associated with the formation of distinct mycorrhizal communities (Davison et al. 2021; Snyder et al. 2025). Furthermore, the low organic matter content recorded in the soil (1.66%) may also have contributed to the structuring of the arbuscular mycorrhizal fungi community in the Palma Agricultural Center. Organic matter is recognized as a relevant factor for the diversity and abundance of these fungi (Shao et al. 2024; Vázquez-Santos et al. 2025), and its low availability can limit the occurrence of certain taxa. This effect can be observed in the low representation of the genus Acaulospora, whose species are frequently reported with greater abundance in soils with a high organic matter content (Vázquez-Santos et al. 2025).

Regarding olive genotypes, the present study observed significant variability in spore abundance among cultivars for three species: *G. decipiens* sporulated preferentially on Arbequina, *D. globifera* on Coratina, and *G. rosea* on Picual. This result is consistent with the findings of Montes-Borrego et al. (2014) and Meddad-Hamza et al. (2017). This cultivar-specific relationship indicates that the host genotype directly influences the structure of the AMF community, probably mediated by the release of different root exudates by each cultivar, which include organic compounds such as organic acids, sugars, amino acids, and phenols. These substances act in the signaling, growth, and development of specific rhizosphere microorganisms (Chen and Liu 2024; Li et al. 2023). Studies by Miho et al. (2021) demonstrate that the Coratina cultivar is particularly rich in oleuropein aglycones, which may have stimulated the greater presence of *D. globifera* in this cultivar, this hypothesis deserves experimental verification in future studies.

At the community scale, this genotype effect also manifested itself consistently. Multivariate analysis confirmed that cultivar identity was the main explanatory factor for the variation in composition, accounting for 56.2% of the total variability. The confirmation of the homogeneity of the multivariate dispersion indicates that the observed differences reflect real changes in community composition, and not statistical artifacts resulting from unequal variation between groups (Anderson 2001). Thus, it was observed that the Coratina cultivar showed greater internal cohesion, indicating that the cultivar may have created a more restrictive ecological filter on the AMF assemblage. These patterns are consistent with recent studies that highlight the determining role of the host genotype in the assembly of AMF communities in perennial agricultural systems under stress conditions (Frew et al. 2025; Mei et al. 2025; Wang et al. 2024).

The analysis revealed that species richness did not differ significantly among cultivars, indicating that AMF are equally accessible to the three olive cultivars. Simpson's index was the only one capable of discriminating cultivars in a statistically robust manner, with Coratina showing significantly lower evenness than Arbequina and Picual, which may be a direct reflection of the disproportionate dominance of *G. ambisporum* in this cultivar. In contrast, Shannon's index showed only a marginal trend indicating greater sensitivity to the observed differences in rare species, and the irregular occurrence of *A. koskei* and *S. calospora* in specific samples of Arbequina and Picual may have increased the internal variance of these cultivars, diluting the differences between groups.

This dominance pattern is consistent with the results showing that the species *G. ambisporum* and *G. decipiens* were primarily responsible for the dissimilarity between Arbequina and Coratina, while *G. rosea* was the main contributor to the difference between Coratina and Picual*.* Compositional differentiation was concentrated in two functionally distinct taxa: a generalist species from the family *Glomeraceae* and a competitive species from the family Gigasporaceae with an extensive extraradical mycelial network (Cahyaningtyas and Ezawa 2024). This result is ecologically relevant because it may indicate that differential signaling between cultivars is not restricted to a single functional group, but encompasses contrasting ecological strategies, suggesting that host-fungus selection mechanisms in olive trees act broadly across the functional spectrum of the community.

The absence of significant differences in species richness among cultivars suggests that the three olive genotypes are associated with a similar regional pool of AMF. However, differences in species abundance and evenness indicate distinct patterns of species dominance, suggesting that the host genotype may influence the relative abundance of particular taxa without substantially altering total species richness pattern, which also observed in olive-associated AMF communities, in studies reported by Meddad-Hamza et al. (2017) and Montes-Borrego et al. (2014). This pattern may partially explain the differences observed in the abundance of *G. decipiens, D. globifera,* and *G. rosea* among cultivars, although additional studies are required to clarify mechanisms that differentiate the community. These differences in community composition may have implications for ecosystem functions associated with the symbiosis. Members of the Glomeraceae family, which dominated all cultivars, are generally recognized for their rapid root colonization and greater tolerance to soil disturbances, whereas representatives of the Gigasporaceae family tend to allocate more biomass to the development of extensive extraradical mycelial networks, potentially contributing to greater soil exploration and nutrient acquisition (Arcidiacono et al. 2024; Cahyaningtyas and Ezawa 2024). Thus, the predominance of functionally contrasting taxa among cultivars may indicate differences in the functional attributes of AMF communities.

From an ecological perspective, differences in community composition among cultivars may potentially influence ecosystem functions associated with root colonization, nutrient acquisition, phosphorus cycling, soil exploration, and plant responses to environmental stresses (Rui et al. 2022). In addition, the identification of autochthonous AMF has agronomic relevance, since olive trees naturally benefit from this symbiosis for adaptation to low-fertility soils and adverse environmental conditions (Chenchouni et al. 2020; Robă et al. 2024; Tekaya et al. 2022). The species identified in the present study, such as *G. ambisporum, D. globifera,* and *G. decipiens*, together with the coexistence of different functional groups, may represent a promising source of propagules for future inoculation programs and sustainable orchard management. Generalist members of the Glomeraceae family are typically associated with rapid root colonization, whereas representatives of the Gigasporaceae family may contribute to greater soil exploration through extensive extraradical mycelial networks, potentially enhancing nutrient acquisition (Arcidiacono et al. 2024; Cahyaningtyas and Ezawa 2024). Thus, differences in these functional attributes among species could potentially influence plant–fungus interactions and the functioning of the symbiosis. However, these interpretations remain speculative, and additional experimental studies are required to determine whether the compositional differences observed among cultivars translate into differences in plant performance and ecosystem services associated with the symbiosis.

Importantly, no commercial AMF inoculants had been applied to the experimental orchard before or during the study period, indicating that the community characterized here was likely predominantly autochthonous. This aspect is ecologically relevant because the introduction of commercial isolates may alter the structure of native AMF communities. Previous studies have shown that introduced fungi can initially suppress the colonization of indigenous AMF, whereas long-term observations indicate that many introduced isolates gradually decline in abundance or are eventually excluded from the system, probably due to the greater ecological adaptation of native communities to local soil and climatic conditions (Basiru and Hijri 2022; Janoušková et al. 2017). In addition to posing a lower risk of ecological imbalance, native AMF frequently exhibit greater persistence and effectiveness under field conditions, suggesting that their use as propagules represents a promising strategy for the development of inoculants adapted to regional edaphoclimatic conditions (Basiru and Hijri 2022).

However, it is important to consider several methodological limitations of the present study. Although previous studies have demonstrated that climatic conditions and soil physicochemical properties are key drivers of AMF community composition (Davison et al. 2021; Meddad-Hamza et al. 2017; Montes-Borrego et al. 2014), these variables were not directly assessed in this work. Therefore, the hypothesis that environmental factors contributed to the prevalence of certain species should be interpreted with caution, requiring further studies to understand these interactions in this cultivation area. In addition, the study was limited to four trees per cultivar due to the size of the experimental area, which restricts the statistical power of the analyses and the generalizability of the results. Although the multivariate analyses applied are appropriate for the sample size, the limited number of replicates may have increased uncertainty, particularly for species with low frequency of occurrence. The identification was based exclusively on spore morphology, which captures only a fraction of the true diversity of AMF present in soil, since taxa that sporulate poorly under field conditions or exhibit cryptic diversity remain undetected (Palla et al. 2020; Stockinger et al. 2010). Consequently, some of the observed patterns in species abundance and composition may reflect sampling and methodological limitations rather than purely ecological processes. Nevertheless, morphology-based identification relying on spore wall structure and differential staining with Melzer’s reagent remains a well-established method in AMF ecology (Melloni et al. 2020; Oehl et al. 2011). Thus, the fungal assemblage described herein provides an important ecological baseline for arbuscular mycorrhizal fungal communities associated with olive cultivation in southern Brazil, a region for which no previous inventories are available. Future studies integrating greater sampling effort, root colonization assessments, molecular approaches, and direct evaluation of environmental variables, as proposed by Kemmelmeier et al. (2022) and Palla et al. (2020), will be necessary to disentangle the relative contributions of environmental conditions, plant genotypes, and sampling effects on the structure of AMF communities associated with olive trees in this region.

## Conclusion

This study presents the first inventory of AMF associated with olive trees in southern Brazil, documenting six species from five genera and four families, representing distinct functional groups previously reported in olive cultivation systems. Four species: *G. ambisporu*m, *G. decipien*s, *G. rosea*, and *D. globifera*, were present in all sampling units and can be characterized as stable and generalist components of the AMF community associated with olive cultivation in the region, regardless of the cultivar. This work establishes an initial ecological baseline for future studies to complement the understanding of AMF-olive interactions and to subsequently support the production of regionally adapted mycorrhiza-based bioinputs.

Taken together, the results suggest that AMF communities associated with olive trees are composed of stable and cultivar-shared taxa, whereas differences in the abundance of some species indicate that host genotype may contribute to shaping community composition. The predominance of members of the Glomeraceae family and their coexistence with representatives of the Gigasporaceae suggest the presence of functionally contrasting groups within the system, highlighting the potential of genera such as *Glomus* and *Gigaspora* for future studies on symbiotic efficiency and the development of regionally adapted inoculants.

However, given the associative nature of the evidence and the methodological limitations of the present study, these patterns require confirmation through studies with greater replication, temporal sampling, and molecular characterization. Further studies are also needed to experimentally evaluate the functional role of these taxa in olive tree growth and nutrition, as well as to investigate the temporal dynamics and broader taxonomic diversity of AMF communities associated with olive cultivation in southern Brazil.

## Supplementary Information

Below is the link to the electronic supplementary material.

Supplementary file1 (XLSX 7 KB)

## Data Availability

Yes. The datasets generated and/or analyzed during the current study are available from the corresponding author on reasonable request.
